# Association of the Total White Blood Cell, Neutrophils, and Monocytes Count With the Presence, Severity, and Types of Carotid Atherosclerotic Plaque

**DOI:** 10.3389/fmed.2020.00313

**Published:** 2020-07-21

**Authors:** Yanhua Liu, Yongjian Zhu, Wenrui Jia, Dan Sun, Li Zhao, Chen Zhang, Cuicui Wang, Quanjun Lyu, Yuming Chen, Gaiyun Chen, Yacong Bo, Yurong Xing

**Affiliations:** ^1^Department of Nutrition, The First Affiliated Hospital of Zhengzhou University, Zhengzhou, China; ^2^Department of Cardiology, The First Affiliated Hospital of Zhengzhou University, Zhengzhou, China; ^3^Department of Physical Center, The First Affiliated Hospital of Zhengzhou University, Zhengzhou, China; ^4^Guangdong Provincial Key Laboratory of Food, Nutrition and Health, School of Public Health, Sun Yat-sen University, Guangzhou, China

**Keywords:** atherosclerosis, carotid atherosclerotic plaque, white blood cell count, neutrophils, monocytes

## Abstract

**Background:** Previous studies have indicated that white blood cells (WBCs) might contribute to the development of atherosclerosis. However, the associations of WBCs and WBC subgroups with carotid atherosclerotic plaque (CAP) have not been compared.

**Methods:** A cross-sectional study including 3,569 healthy Chinese adults was conducted between January 2016 and December 2018 in Zhengzhou, China, to explore the associations of WBC and WBC subtypes with the presence, severity, and types of CAPs. Fasting peripheral venous blood was collected for measurement of the total WBC and WBC subtype counts. The size, composition, and types of CAPs in the common carotid artery, the internal carotid artery, and the external carotid artery were measured bilaterally using B-mode ultrasound.

**Results:** The total WBC, neutrophil, and monocyte counts showed significant associations with the presence of CAPs in men, but not in women, with the adjusted odds ratios (95% CI) in the highest (compared to the lowest) quartile 1.99 (1.33–2.97), 1.65 (1.10–2.47), and 2.17 (1.41–3.18) (*P*_trend_ = 0.004, *P*_trend_ = 0.004, and *P*_trend_ < 0.001), respectively. The three leukocyte counts were also significantly associated with the severity of CAPs, as judged by the count of CAPs, maximal internal carotid plaque thickness, and the plaque score (all *P* < 0.01, *P*_trend_ < 0.05). Compared with individuals without CAPs, those with echolucent plaques had significantly increased total WBC and neutrophil counts, whereas those with polytype plaques had a significantly increased monocyte count.

**Conclusion:** WBC, neutrophil, and monocyte counts were significantly associated with the presence, severity, and types of CAPs in a healthy Chinese population.

## Introduction

Atherosclerosis is a strong risk factor for cardiovascular and cerebrovascular diseases, which are the leading causes of death and disability worldwide (1), and have become an important public health concern in China (2). Although individuals with atherosclerosis are usually without symptoms in the early stages, atherosclerosis can progress at their young age and throughout the lifetime. Atherosclerosis involves the progressive narrowing of the vessel's lumens, which may lead to acute cardiac ischemia, myocardial infarction, or stroke. The carotid intima–media thickness (CIMT), measured by a carotid ultrasound, has been used as a surrogate indicator for atherosclerosis. However, CIMT provided less clear evidence for atherosclerotic infiltration in the vessel wall than a significant carotid atherosclerotic plaque (CAP) does (3). Several studies have demonstrated that, compared to the mean IMT, CAP could predict the occurrence of cardiovascular and cerebrovascular diseases more accurately.

Recently, it has been recognized that atherogenesis is not only a passive injury with infiltration of lipids but is also an active inflammatory process. The relationship of the white blood cell (WBC) count with inflammation in various diseases has been investigated (4–6). WBCs comprised the subtypes of neutrophils, lymphocytes, monocytes, eosinophils, and basophils, each of which plays a distinct inflammatory and immunologic role and may contribute differently to the development of atherosclerosis (7). Several studies have confirmed the association of the WBC count with the risk of cardiac cerebral disease (8, 9). Recruitment of leukocytes to the vascular endothelium wall represents an early stage in the initiation and development of atherosclerosis (10). Although increased leukocytes have been reported to correlate with CAP, the associations of the WBC subgroups with CAPs have not been compared (11). As the WBC count is an inexpensive and widely used test, we conducted the current study to more fully explore the associations between WBCs and the presence, severity, and types of CAPs.

## Methods

### Design and Study Population

This cross-sectional study included 3,569 healthy Chinese subjects (2,464 men and 1,105 women) aged 18–97 years, and the associations of the total WBC and WBC subtypes with CAPs were investigated. All subjects were recruited through a general health screening programme between January 2016 and December 2018 in Zhengzhou, China. Individuals were excluded for the following reasons: (i) a history of cardiovascular disease, including coronary heart disease, stroke, thromboembolic disease, congestive heart failure, or peripheral arterial disease; (ii) cancer; (iii) infectious disease; (iv) serious liver or renal disorders; and (v) currently on medication. The current study was approved by the Research Ethical Committee of the First Affiliated Hospital of Zhengzhou University, and written informed consent was obtained from each participant at the time of enrollment.

### Parameter Measurements

Weight and height were measured using an autoanthropometer, and body mass index (BMI) was calculated with the formula: weight in kilograms divided by height in meter squared. Blood pressure was measured by an electronic sphygmomanometer after the subjects had been sitting in a relaxed position for 10 min. Data on smoking, drinking, and history of diseases were collected using a standard physician-administered questionnaire. Smokers and drinkers were defined as participants who smoked one or more cigarettes per day and drank alcohol once or more a week for at least 6 months, respectively.

Fasting peripheral vein blood (5 ml) was drawn in EDTA-anticoagulant tubes. After centrifugation at 1,500 × g for 15 min at 4°C, plasma and blood cells were separated within 2 h. The total WBC and WBC subtype (neutrophils, lymphocytes, monocytes, eosinophils, and basophils) counts and their percentages were assayed automatically by a blood cell counter (Beckman Coulter, Miami, FL, USA). Standard quality control procedures were followed in the laboratory. The plasma levels of total cholesterol (TC), triglycerides (TG), high-density lipoprotein cholesterol (HDLc), low-density lipoprotein cholesterol (LDLc), glucose, and uric acid were measured using a fully automatic analyzer (Roche Molecular Systems, Inc., Basel, Switzerland) module Cobas 8000 (C701/C702/C502). The coefficient of variation for repeated measurements on the blood samples was maintained at a level of 2.5%.

### Atherosclerosis Assessment

The presence of CAPs was measured bilaterally by B-mode ultrasound using an Aplio 300 ultrasound system (Toshiba Medical Systems, Tokyo, Japan). Measurements were taken in the far wall of the common carotid artery (CCA), the internal carotid artery (ICA), and the external carotid artery (ECA). When the subjects have lain in a supine position, the image of the extracranial carotid arteries were acquired in the longitudinal (anterior, lateral, and posterior views) and transverse planes. CAP was defined as a local protrusion of at least 0.5 mm, a 50% local thickening of IMT, or a CIMT >1.5 mm (12).

When a plaque was imaged, the view of the thickest part of the plaque was frozen and an electronic cursor was used to measure the intimal–medial wall thickness (including the plaque), recording it as the maximal internal carotid plaque thickness (MICPT) for that artery. When no plaque was identified, the MICPT was recorded as zero (13). The plaque score was thereafter calculated by summing the measurements of plaque thickness in both carotid arteries (14).

According to ultrasound imaging, plaques were classified into three types: echolucent (low grayscale median, lipid-rich), echogenic (high grayscale median, mostly occupied by calcified areas), or heterogeneous (mixed echolucent and echogenic) (15). A plaque was classified as a polytype when more than one composition was present. All CAP measurements were conducted by the same senior physician using B-mode ultrasound, and strict quality control procedures were followed during image acquisition.

### Statistical Analyses

The data are presented as means with standard deviations (SDs) for the continuous variables and count with frequencies for the categorical variables. Student's *t*-test and Pearson's chi-square test were conducted, as appropriate. Pearson's correlation analysis was used to assess the relationship between blood cell counts and lipid profiles.

The study participants were divided into quartiles according to their total and differential WBC counts and by gender. Binary logistical regression models were applied to estimate the association of total and differential WBC counts with the presence of CAPs. Three models were developed: model 1, adjusted for age and BMI; model 2, further adjusted for fasting plasma lipid profiles, fasting plasma glucose, systolic blood pressure (SBP), diastolic blood pressure (DBP), and uric acid; and model 3, further adjusted for drinker and smoker (only for men). The potential non-linear associations of the blood cell counts with the presence of CAPs were assessed by restricted cubic spline with three knots.

The study participants were also divided into quartiles according to their MICPT and plaque scores. The lowest two quartiles (Q1 and Q2) were combined into one category (Q1–Q2); the MICPT and plaque scores of all participants in these quartiles were recorded as zero. Analysis of covariance (ANCOVA) was adopted to compare the mean total and differential WBC counts according to the sum and the composition of CAPs and the quartiles of MICPT and plaque score. The same covariates were adjusted for the ANCOVA as those in the regression analysis. Pairwise comparisons were performed using the Bonferroni test.

The analyses were performed with SPSS 13.0 (SPSS, Inc., Chicago, USA) and R 3.6.0 (R Core Team, Vienna, Austria). Two-sided *P* < 0.05 indicated statistical significance.

## Results

### Baseline Characteristics

Of the 3,569 subjects, 331 were excluded in accordance with the exclusion criteria and due to incomplete data. Thus, a total of 3,238 participants were included in the analysis. Table 1 summarizes the baseline characteristics of the study participants by gender. The 2,249 men and 989 women had mean ages of 50.6 ± 10.8 and 51.2 ± 10.8 years, respectively. Total WBC and most of the differential WBC counts were higher in men than in women (*P* ≤ 0.010). The percentages of subjects with plaques in any carotid artery, CCA, ICA, or ECA were also higher in men than in women (*P* < 0.001): 49.6, 46.6, 14.2, and 3.7% for men and 31.9, 29.0, 6.5, and 1.4% for women, respectively. Sex differences were also found in the MICPT, plaque score, and the count and composition of CAPs (*P* < 0.001). Pearson's analysis revealed that the total WBC and neutrophil counts were significantly associated with TG and HDL-c (*P* < 0.05), but not TC or LDL-c, and the monocyte count was only significantly associated with TG (*P* < 0.05; Figure S1).

**Table 1:** Characteristics of the study participants.

	**Men**			**Women**			***P***
	**Mean**	***SD***	***N***	**Mean**	***SD***	***N***	
Age (years)	50.6	10.8	2,249	51.2	10.8	989	0.154
Height (cm)	172.5	5.7	1,948	160.7	5.9	824	<0.001
Weight (kg)	77.8	10.0	1,949	62.1	8.8	826	<0.001
BMI (kg/m^2^)	26.1	2.9	1,948	24.0	3.2	824	<0.001
SBP (mmHg)	132.6	17.7	1,934	127.3	19.8	808	<0.001
DBP (mmHg)	82.2	12.9	1,933	74.4	12.8	808	<0.001
Plasma glucose (mmol/L)	5.40	1.62	2,147	5.16	1.15	925	<0.001
Uric acid (mmol/L)	348.9	91.5	2,197	253.4	75.2	975	<0.001
TC (mmol/L)	4.67	1.07	2,126	4.73	1.05	959	0.158
TG (mmol/L)	2.05	4.57	2,146	1.42	0.96	961	<0.001
HDLc (mmol/L)	1.30	0.47	2,143	1.50	0.46	953	<0.001
LDLc (mmol/L)	2.95	0.87	2,101	2.99	0.81	931	0.212
Total WBC Count (× 10^9^/L)	6.44	1.68	2,236	5.82	1.55	987	<0.001
Neutrophils (× 10^9^/L)	3.79	1.53	2,248	3.38	1.16	989	<0.001
Neutrophils (%)	57.7	7.74	2,245	57.5	7.90	988	0.555
Lymphocytes (× 10^9^/L)	2.04	0.58	2,247	1.90	0.57	986	<0.001
Lymphocytes (%)	32.0	7.07	2,221	33.3	7.16	981	<0.001
Monocytes (× 10^9^/L)	0.46	0.24	2,243	0.38	0.25	988	<0.001
Monocytes (%)	7.09	1.83	2,245	6.49	1.63	989	<0.001
Eosinophils (× 10^9^/L)	0.43	1.17	2,247	0.33	0.86	988	0.01
Eosinophils (%)	2.34	2.25	2,248	1.94	1.86	988	<0.001
Basophils (× 10^9^/L)	0.05	0.08	2,246	0.05	0.08	989	0.286
Basophils (%)	0.52	0.25	2,248	0.53	0.22	989	0.302
MICPT (mm)	1.04	1.14	2,249	0.99	0.61	989	<0.001
Plaque score (mm)	2.18	3.10	2,249	1.10	2.12	989	<0.001
	***n***	**%**	***N***	***n***	**%**	***N***	***P***
Smoking	60	4.7	1,273	0	0.0	515	<0.001
Drinking	68	5.3	1,205	1	0.2	514	<0.001
Carotid plaque	1,115	49.6	2,249	315	31.9	989	<0.001
Plaque at CCA	1,047	46.6	2,249	287	29.0	989	<0.001
Plaque at ICA	320	14.2	2,249	64	6.5	989	<0.001
Plaque at ECA	84	3.7	2,249	14	1.4	989	<0.001
**Carotid plaque**							<0.001
**composition**							
None	1,134	50.4	2,249	674	68.1	989	
Echolucent	440	19.6	2,249	125	12.6	989	
Echogenic	13	0.6	2,249	10	1.0	989	
Heterogeneous	307	13.7	2,249	106	10.7	989	
Polytype	355	15.8	2,249	74	7.5	989	
**Count of carotid plaque**							<0.001
0	1,134	50.0	2,249	674	68.1	989	
1	441	19.6	2,249	159	16.1	989	
2	279	12.4	2,249	83	8.4	989	
≥3	394	17.5	2,249	72	7.3	989	

Plaque score is the sum of all the plaque thickness measurements. Carotid plaque composition: if all the plaques in a subject had the same composition, the subject was classified according to that composition; if the plaque had different composition, the subject was classified as having polytype plaques.

*BMI, body mass index; SBP, systolic blood pressure; DBP, diastolic blood pressure; TC, total cholesterol; TG, triglycerides; HDLc, high-density lipoprotein cholesterol; LDLc, low-density lipoprotein cholesterol; WBC, white blood cell; MICPT, maximal internal carotid plaque thickness; CCA, common carotid artery; ICA, internal carotid artery; ECA, external carotid artery*.

### WBC and the Presence of CAPs

In men, the total WBC, neutrophil, and monocyte counts showed significant positive correlations with the presence of CAPs after adjustment for age, BMI, SBP, DBP, TC, TG, HDLc, LDLc, fasting glucose, and uric acid (*P* < 0.001, *P*_trend_ < 0.001; Table 2). In women, the total WBC and differential WBC counts showed no significant association with the presence of CAPs (Table 3). The associations of the total WBC, neutrophil, and monocyte counts with the presence of CAPs existed even after further adjustment for smoking and drinking. The odds ratios (95% CI) of the individuals with CAP in the highest (compared to the lowest) quartile were 1.99 (1.33–2.97), 1.65 (1.10–2.47), and 2.17 (1.41–3.18) (*P*_trend_ = 0.004, *P*_trend_ = 0.004, and *P*_trend_ < 0.001, respectively) for the total WBC, neutrophil, and monocytes count, respectively. Similar, but less robust, results were found for the percentages of neutrophils, lymphocytes, and basophils (*P* < 0.05, *P*_trend_ < 0.05). Further analysis revealed that the significant associations of the total WBC, neutrophil, and monocyte counts with the presence of CAPs only existed in men aged 45–65 years (Tables 4, 5). Additionally, restrictive cubic spline showed that the total WBC, neutrophil, and monocyte counts had a non-linear relationship with the presence of CAPs, consistent with the main results (Figures S2–S4).

**Table 2:** Odds ratios and 95% CI for carotid artery plaque by quartiles of the total white blood cell (WBC) and differential WBC counts for men.

	**Odds ratios (95% CI) for carotid plaque**				
	**Q1**	**Q2**	**Q3**	**Q4**	***P*_**trend**_**
**Total WBC count (× 10**^**9**^**/L)**					
Cases, *N*	472	485	504	475	
Model 1	1.00	1.30 (0.98–1.73)	1.22 (0.92–1.62)	2.06 (1.54–2.76)^***^	**<0.001**
Model 2	1.00	1.31 (0.96–1.80)	1.21 (0.89–1.65)	2.07 (1.50–2.85)^***^	**<0.001**
Model 3	1.00	1.56 (1.05–2.31)^*^	1.36 (0.92–2.02)	1.99 (1.33–2.97)^**^	**0.004**
**Neutrophils (× 10**^**9**^**/L)**					
Cases, *N*	456	517	481	493	
Model 1	1.00	1.39 (0.99–1.76)	1.60 (1.20–2.14)^**^	1.99 (1.49–2.66)^***^	**<0.001**
Model 2	1.00	1.38 (1.01–1.89)^*^	1.76 (1.28–2.42)^***^	2.10 (1.52–2.89)^***^	**<0.001**
Model 3	1.00	1.39 (0.95–2.06)	1.97 (1.33–2.93)^**^	1.65 (1.10–2.47)^*^	**0.004**
**Neutrophils (%)**					
Cases, *N*	481	493	498	473	
Model 1	1.00	1.53 (1.15–2.03)^**^	1.46 (1.10–1.93)^**^	1.36 (1.02–1.81)^*^	0.063
Model 2	1.00	1.50 (1.11–2.04)^**^	1.65 (1.21–2.24)^**^	1.38 (1.01–1.89)^*^	**0.031**
Model 3	1.00	1.26 (0.86–1.83)	1.58 (1.08–2.31)^*^	0.97 (0.64–1.45)	0.696
**Lymphocytes (× 10**^**9**^**/L)**					
Cases, *N*	525	424	494	504	
Model 1	1.00	1.00 (0.75–1.33)	1.05 (0.79–1.38)	1.48 (1.12–1.95)^**^	**0.007**
Model 2	1.00	0.91 (0.66–1.25)	1.05 (0.77–1.42)	1.36 (1.00–1.85)^*^	**0.033**
Model 3	1.00	0.88 (0.59–1.32)	0.98 (0.65–1.40)	1.41 (0.96–2.08)	0.076
**Lymphocytes (%)**					
Cases, *N*	473	500	468	480	
Model 1	1.00	1.06 (0.80–1.40)	0.94 (0.71–1.25)	0.84 (0.63–1.12)	0.155
Model 2	1.00	1.04 (0.76–1.42)	0.94 (0.68–1.28)	0.75 (0.54–1.02)	**0.047**
Model 3	1.00	1.39 (0.93–2.06)	1.04 (0.69–1.56)	1.09 (0.72–1.63)	0.837
**Monocytes (× 10**^**9**^**/L)**					
Cases, *N*	485	507	484	467	
Model 1	1.00	1.22 (0.93–1.62)	1.20 (0.90–1.59)	2.19 (1.64–2.93)^***^	**<0.001**
Model 2	1.00	1.24 (0.91–1.68)	1.26 (0.92–1.72)	2.14 (1.56–2.94)^***^	**<0.001**
Model 3	1.00	1.46 (0.99–2.17)	1.69 (1.13–2.47)^*^	2.17 (1.41–3.18)^***^	**<0.001**
**Monocytes (%)**					
Cases, *N*	479	493	498	475	
Model 1	1.00	1.13 (0.85–1.49)	1.15 (0.87–1.52)	1.13 (0.85–1.50)	0.418
Model 2	1.00	1.10 (0.81–1.49)	1.17 (0.86–1.59)	1.14 (0.84–1.56)	0.367
Model 3	1.00	1.07 (0.73–1.57)	1.18 (0.80–1.76)	1.28 (0.87–1.89)	0.179
**Eosinophils (× 10**^**9**^**/L)**					
Cases, *N*	531	448	470	497	
Model 1	1.00	1.23 (0.96–1.62)	1.29 (0.97–1.70)	1.31 (1.00–1.73)	**0.049**
Model 2	1.00	1.29 (0.94–1.75)	1.27 (0.93–1.73)	1.35 (0.99–1.82)	0.071
Model 3	1.00	1.51 (1.02–2.23)^*^	1.55 (1.05–2.28)^*^	1.28 (0.88–1.88)	0.200
**Eosinophils (%)**					
Cases, *N*	475	479	504	489	
Model 1	1.00	0.81 (0.61–1.08)	1.00 (0.78–1.32)	0.89 (0.67–1.18)	0.767
Model 2	1.00	0.81 (0.60–1.10)	0.95 (0.70–1.28)	0.86 (0.63–1.17)	0.543
Model 3	1.00	0.92 (0.62–1.35)	1.33 (0.91–1.95)	1.07 (0.73–1.57)	0.359
**Basophils (× 10**^**9**^**/L)**					
Cases, *N*	556	566	389	434	
Model 1	1.00	1.17 (0.90–1.53)	1.35 (1.01–1.81)^*^	1.42 (1.07–1.89)^*^	**0.009**
Model 2	1.00	1.16 (0.88–1.54)	1.45 (1.05–1.99)^*^	1.46 (1.07–2.00)^*^	**0.007**
Model 3	1.00	1.01 (0.70–1.44)	1.21 (0.81–1.82)	1.45 (0.99–2.13)	**0.039**
**Basophils (%)**					
Cases, *N*	333	470	752	392	
Model 1	1.00	0.91 (0.66–1.24)	1.01 (0.75–1.34)	0.99 (0.71–1.37)	0.806
Model 2	1.00	0.88 (0.62–1.24)	1.01 (0.73–1.38)	1.06 (0.74–1.51)	0.515
Model 3	1.00	0.92 (0.59–1.43)	0.86 (0.57–1.30)	1.14 (0.72–1.81)	0.648

Model 1 was adjusted for age and BMI.

Model 2 included additional adjustments for systolic blood pressure, diastolic blood pressure, total cholesterol, triglycerides, high-density lipoprotein cholesterol, low-density lipoprotein cholesterol, fasting plasma glucose, and uric acid.

Model 3 included additional adjustments for drinking and smoking.

* P ≤ 0.05,

** P ≤ 0.01, and

*** *P ≤ 0.001 compared with Q1 (Bonferroni). Bold P indicates statistical significance*.

**Table 3:** Odds ratios and 95% CI for carotid artery plaque by quartiles of total white blood cell (WBC) and differential WBC counts for women.

	**Odds ratios (95% CI) for carotid plaque**				
	**Q1**	**Q2**	**Q3**	**Q4**	***P*_**trend**_**
**Total WBC count (× 10**^**9**^**/L)**					
Cases, *N*	219	184	223	196	
Model 1	1.00	1.20 (0.74–1.97)	1.32 (0.83–2.11)	1.06 (0.65–1.75)	0.677
Model 2	1.00	1.12 (0.65–1.98)	1.12 (0.67–1.88)	0.95 (0.55–1.64)	0.879
**Neutrophils (× 10**^**9**^**/L)**					
Cases, *N*	224	214	182	204	
Model 1	1.00	1.69 (1.05–2.70)	1.35 (0.82–2.23)	1.60 (0.98–2.62)	0.125
Model 2	1.00	1.49 (0.89–2.50)	1.20 (0.69–2.09)	1.43 (0.83–2.45)	0.337
**Neutrophils (%)**					
Cases, *N*	208	208	206	201	
Model 1	1.00	1.13 (0.70–1.82)	0.98 (0.60–1.59)	1.60 (0.99–2.61)	0.114
Model 2	1.00	1.12 (0.66–1.88)	0.97 (0.57–1.65)	1.55 (0.91–2.66)	0.190
**Lymphocytes (× 10**^**9**^**/L)**					
Cases, *N*	227	211	200	185	
Model 1	1.00	0.75 (0.46–1.21)	0.92 (0.57–1.46)	0.81 (0.50–1.32)	0.567
Model 2	1.00	0.79 (0.47–1.33)	0.72 (0.43–1.20)	0.74 (0.43–1.28)	0.239
**Lymphocytes (%)**					
Cases, *N*	193	213	204	206	
Model 1	1.00	0.79 (0.49–1.29)	0.72 (0.44–1.18)	0.67 (0.41–1.10)	0.114
Model 2	1.00	0.66 (0.38–1.13)	0.68 (0.40–1.16)	0.66 (0.38–1.12)	0.160
**Monocytes (× 10**^**9**^**/L)**					
Cases, *N*	114	307	214	188	
Model 1	1.00	1.62 (0.93–2.81)	1.18 (0.65–2.13)	1.41 (0.77–2.58)	0.802
Model 2	1.00	1.37 (0.74–2.54)	1.01 (0.53–1.95)	1.36 (0.71–2.63)	0.746
**Monocytes (%)**					
Cases, *N*	210	211	193	210	
Model 1	1.00	1.02 (0.64–1.64)	0.83 (0.51–1.35)	0.87 (0.54–1.40)	0.417
Model 2	1.00	1.09 (0.65–1.84)	0.87 (0.50–1.49)	0.98 (0.58–1.65)	0.749
**Eosinophils (× 10**^**9**^**/L)**					
Cases, *N*	211	203	214	195	
Model 1	1.00	1.00 (0.61–1.62)	1.04 (0.64–1.68)	0.71 (0.43–1.18)	0.247
Model 2	1.00	0.73 (0.42–1.28)	1.03 (0.60–1.76)	0.67 (0.38–1.18)	0.366
**Eosinophils (%)**					
Cases, *N*	206	200	215	203	
Model 1	1.00	1.33 (0.82–2.16)	0.94 (0.58–1.53)	0.84 (0.51–1.38)	0.275
Model 2	1.00	1.05 (0.62–1.80)	0.81 (0.48–1.39)	0.81 (0.47–1.38)	0.293
**Basophils (× 10**^**9**^**/L)**					
Cases, *N*	271	266	138	149	
Model 1	1.00	0.79 (0.52–1.19)	0.70 (0.42–1.16)	0.68 (0.41–1.13)	0.091
Model 2	1.00	0.74 (0.47–1.18)	0.68 (0.39–1.17)	0.73 (0.41–1.29)	0.165
**Basophils (%)**					
Cases, *N*	128	368	146	182	
Model 1	1.00	1.24 (0.76–2.03)	0.74 (0.40–1.35)	0.82 (0.46–1.44)	0.116
Model 2	1.00	1.36 (0.79–2.33)	0.78 (0.40–1.50)	0.97 (0.52–1.81)	0.345

Model 1 was adjusted for age and BMI.

*Model 2 included additional adjustments for systolic blood pressure, diastolic blood pressure, total cholesterol, triglycerides, high-density lipoprotein cholesterol, low-density lipoprotein cholesterol, fasting plasma glucose, and uric acid*.

**Table 4:** Adjusted odds ratios and 95% CI for carotid artery plaque stratified by age for men.

	**Adjusted odds ratios (95% CI) for carotid plaque**				
	**Q1**	**Q2**	**Q3**	**Q4**	***P*_**trend**_**
**Total WBC count (× 10**^**9**^**/L)**					
<45 years	1.00	1.50 (0.64–3.53)	1.64 (0.72–3.70)	1.85 (0.83–4.10)	0.170
45–65 years	1.00	1.16 (0.81–1.65)	1.11 (0.78–1.58)	1.82 (1.28–2.59)^***^	**<0.001**
≥65 years	1.00	1.80 (0.46–7.09)	0.70 (0.18–2.71)	1.74 (0.44–6.92)	0.479
*P*_interaction_	0.807				
**Neutrophils (× 10**^**9**^**/L)**					
<45 years	1.00	2.44 (0.96–6.20)	2.96 (1.17–7.48)^*^	3.18 (1.26–8.03)^*^	0.071
45–65 years	1.00	1.26 (0.88–1.81)	1.59 (1.12–2.25)^**^	1.93 (1.35–2.78)^***^	**0.012**
≥65 years	1.00	1.32 (0.35–5.02)	3.46 (0.73–16.41)	1.55 (0.41–5.93)	0.218
*P*_interaction_	0.333				
**Lymphocytes (× 10**^**9**^**/L)**					
<45 years	1.00	0.90 (0.39–2.08)	0.65 (0.28–1.53)	1.15 (0.52–2.55)	0.725
45–65 years	1.00	0.99 (0.69–1.41)	1.12 (0.78–1.62)	1.41 (0.98–2.05)	**0.047**
≥65 years	1.00	0.57 (0.16–2.07)	1.22 (0.25–5.85)	0.58 (0.15–2.34)	0.274
*P*_interaction_	0.426				
**Monocytes (× 10**^**9**^**/L)**					
<45 years	1.00	1.60 (0.76–3.36)	0.87 (0.41–1.85)	1.31 (0.62–2.77)	0.770
45–65 years	1.00	1.15 (0.82–1.62)	1.56 (1.09–2.23)^*^	2.29 (1.59–3.30)^***^	**<0.001**
≥65 years	1.00	3.29 (0.78–13.78)	0.99 (0.28–3.49)	24.40 (2.48–239.75)^**^	0.104
*P*_interaction_	0.341				
**Eosinophils (× 10**^**9**^**/L)**					
<45 years	1.00	1.63 (0.75–3.52)	1.70 (0.80–3.60)	1.28 (0.58–2.81)	0.602
45–65 years	1.00	1.06 (0.73–1.53)	1.16 (0.82–1.64)	1.23 (0.87–1.75)	0.584
≥65 years	1.00	2.53 (0.66–9.67)	2.41 (0.68–8.51)	7.13 (1.61–31.64)	0.394
*P*_interaction_	0.911				
**Basophils (× 10**^**9**^**/L)**					
<45 years	1.00	1.56 (0.77–3.15)	1.44 (0.68–3.07)	1.01 (0.47–2.17)	0.711
45–65 years	1.00	1.01 (0.74–1.37)	1.47 (1.03–2.08)^*^	1.63 (1.14–2.32)^**^	0.921
≥65 years	1.00	10.94 (1.19–100.27)^*^	2.02 (0.51–8.01)	2.09 (0.67–6.54)	0.675
*P*_interaction_	0.884				

Model adjusted for BMI, systolic blood pressure, diastolic blood pressure, total cholesterol, triglycerides, high-density lipoprotein cholesterol, low-density lipoprotein cholesterol, fasting plasma glucose, and uric acid.

WBC, white blood cell.

* P ≤ 0.05,

** P ≤ 0.01, and

*** *P ≤ 0.001 compared with Q1 (Bonferroni). Bold P indicates statistical significance*.

**Table 5:** Adjusted odds ratios and 95% CI for carotid artery plaque stratified by age for women.

	**Adjusted odds ratios (95% CI) for carotid plaque**				
	**Q1**	**Q2**	**Q3**	**Q4**	***P*_**trend**_**
**Total WBC count (× 10**^**9**^**/L)**					
<45 years	1.00	0.15 (0.01–1.76)	0.88 (0.16–5.00)	0.16 (0.01–2.07)	0.263
45–65 years	1.00	1.02 (0.60–1.72)	1.05 (0.60–1.85)	0.89 (0.46–1.73)	0.656
≥65 years	1.00	0.81 (0.19–3.42)	0.87 (0.16–4.63)	0.39 (0.09–1.65)	0.206
*P*_interaction_	0.111				
**Neutrophils (× 10**^**9**^**/L)**					
<45 years	1.00	1.19 (0.19–7.30)	1.23 (0.20–7.40)	0.18 (0.01–2.37)	0.149
45–65 years	1.00	1.23 (0.71–2.12)	1.30 (0.72–2.37)	1.94 (1.02–2.67)	0.510
≥65 years	1.00	0.64 (0.15–2.75)	0.80 (0.15–4.24)	0.27 (0.06–1.18)	0.366
*P*_interaction_	0.168				
**Lymphocytes (× 10**^**9**^**/L)**					
<45 years	1.00	0.35 (0.05–2.50)	2.27 (0.40–2.51)	0.54 (0.05–2.51)	0.757
45–65 years	1.00	0.76 (0.44–1.30)	0.71 (0.39–1.29)	0.98 (0.52–1.83)	0.440
≥65 years	1.00	1.31 (0.26–6.61)	0.66 (0.14–2.99)	0.51 (0.11–2.29)	0.520
*P*_interaction_	0.471				
**Monocytes (× 10**^**9**^**/L)**					
<45 years	1.00	2.20 (0.34–14.47)	8.26 (1.28–53.25)^*^	–	0.921
45–65 years	1.00	0.83 (0.50–1.36)	1.12 (0.62–2.04)	0.85 (0.38–1.92)	0.703
≥65 years	1.00	0.56 (0.15–2.07)	0.73 (0.14–3.86)	0.26 (0.04–1.63)	0.164
*P*_interaction_	0.235				
**Eosinophils (× 10**^**9**^**/L)**					
<45 years	1.00	0.52 (0.08–3.39)	1.22 (0.22–6.63)	–	0.378
45–65 years	1.00	1.02 (0.57–1.81)	1.43 (0.81–2.53)	1.02 (0.54–1.93)	0.728
≥65 years	1.00	0.48 (0.09–2.71)	0.50 (0.09–2.85)	0.90 (0.15–5.53)	0.314
*P*_interaction_	0.066				
**Basophils (× 10**^**9**^**/L)**					
<45 years	1.00	0.52 (0.09–2.91)	0.26 (0.02–2.94)	0.67 (0.10–2.91)	0.626
45–65 years	1.00	0.88 (0.53–1.46)	0.82 (0.45–1.51)	0.73 (0.38–1.41)	0.308
≥65 years	1.00	0.14 (0.03–0.75)^*^	0.16 (0.03–0.93)^*^	0.22 (0.04–1.26)	0.470
*P*_interaction_	0.435				

Model adjusted for BMI, systolic blood pressure, diastolic blood pressure, total cholesterol, triglycerides, high-density lipoprotein cholesterol, low-density lipoprotein cholesterol, fasting plasma glucose, and uric acid.

WBC, white blood cell.

* *P ≤ 0.05 compared with Q1 (Bonferroni)*.

### WBCs and the Severity of CAPs

In the total study population, the total WBC, neutrophil, and monocyte counts were significantly correlated with the severity of carotid atherosclerosis, as judged by either the sum of CAPs, MICPT, or the plaque score (all *P* < 0.01, *P*_trend_ < 0.05; Tables S1–S3). Similar results were found for the percentage of lymphocytes (*P* < 0.05).

### WBCs and the Types of CAPs

Significant differences were observed between participants without CAPs and participants with different subtypes of CAPs (Table S4). Compared with the participants without CAPs, those with echolucent plaques showed significantly increased total WBC and neutrophil counts, but significantly decreased percentage of lymphocytes, whereas those with polytype plaques showed significantly increased monocyte counts.

## Discussion

To our knowledge, the current study is the first to investigate the associations of the total WBC and WBC subtype counts with the presence, severity, and subtypes of CAPs in a healthy Chinese population. Our results suggest that the total WBC and WBC subtype counts contain important risk information for CAPs. The total WBC, neutrophil, and monocyte counts were significantly associated with increased odds of the presence of CAPs in men, but not in women after adjustment for potential confounding factors. These three counts also showed significant positive associations with the severity and subtypes of CAPs.

Several epidemiologic observational studies have reported that an increased WBC count is associated with a higher cardiovascular risk prediction (8, 9). The total WBC and neutrophil counts are also confirmed as risk factors for the outcomes of myocardial infarction and cerebral infarction (16, 17) and are associated with infarction size (18). Interestingly, the neutrophil count has been found to be significantly associated with the angiographic characteristics of coronary artery disease, strongly suggesting that neutrophil was involved in the development of coronary atherosclerosis (19). The results of our study not only confirm the positive relationship between the total WBC count and CAPs but also show that neutrophils and monocytes are superior to other WBC subtypes in indicating the presence of CAPs.

Gender differences in the prevalence and complications of atherosclerosis are well-known. However, data are limited in the clinical and preclinical literature to provide robust evidence regarding the underlying mechanisms. Genetic background and biological heterogeneity might play essential roles in the gender difference observed in atherosclerosis (20). Moreover, the influence of sex hormones on atherosclerosis is still controversial (21). Research indicates that although younger women have a lower risk of cardiovascular disease than men, women's risk catches up to and even surpasses that of men at the age of 60–79 years (22). Moreover, the risk factors for atherosclerosis might differ between genders. In our study, the overall BMI, blood pressure, and lipid profiles were significantly lower in women, which might have contributed to the differential results in the association between leukocytes and CAPs.

Studies have shown that CAP burden might be an independent indicator of the severity of intracranial artery atherosclerosis and can also predict future cardiovascular and cerebrovascular events (23). Hence, CAP burden may be more meaningful than just the presence or absence of a carotid plaque. A study among Hispanics found that individuals in the highest quartile of the WBC count had significantly increased MICPT compared with the subjects in the lowest quartile (13). Another study in non-smoking men (*n* = 571) found that the presence and severity of carotid atherosclerosis were significantly associated with increased counts of total WBCs, neutrophils, and monocytes (24). These results are consistent with our finding that the total WBC, neutrophil, and monocyte counts were significantly positively correlated with the sum of CAPs, MICPT, and the plaque score.

The risk of rupture of atherosclerotic plaques is a main concern in carotid atherosclerosis. Generally, the stability of CAP could be indicated by its compositions. Echolucent or hypoechogenic plaques were mainly composed of lipids, inflammatory cells, and neovessels. However, echogenic plaques mainly comprise calcified tissues (25). Previous studies have found that compared to echogenic plaques, echolucent plaques are associated with cerebrovascular events and future coronary events (26). The leukocyte count has been reported to be associated with the degree of CAP instability (27), and a high neutrophil count in CAPs is related to the features of rupture-prone lesions (28). In our study, we also found that, compared with the participants without CAP, those with echolucent plaques showed significantly increased total WBC and neutrophils counts, whereas those with polytype plaques showed significantly increased monocyte counts.

Several pathophysiological mechanisms potentially link the WBCs and WBC subtypes to plaque formation and vulnerability. First of all, recruitment of leukocytes to the vessel endothelium wall represents an early stage in the development of atherosclerosis. CAP is characterized by the infiltration of inflammatory cells, which transmigrated from blood into the subendothelial layers of the arteries (29). Monocytes are the primary inflammatory infiltrating cells in early atherosclerotic plaques, and their accumulation increased proportionally with plaque size (30). The presence of neutrophil invasion in atherosclerotic plaques has been shown in an animal model and in patients with acute coronary syndrome (31). Neutrophils localized in rupture-prone areas and released active mediators, which caused plaque instability (28). Secondly, inflammation cells play a vital role in the progress of atherosclerosis and plaque vulnerability (32). Monocytes, as representatives of the innate immune system, could drive the progression of atherosclerosis from a stable to an unstable state (33, 34). Neutrophils also promoted the progression of atherosclerosis because of their ability to activate other immune cells, especially monocytes, instructing them to directly participate in the inflammation, and/or release pro-inflammatory mediators. Furthermore, neutrophils might increase plaque vulnerability by releasing various enzymes, including gelatinase, collagenase, myeloperoxidase, and elastase (35).

This study has some limitations to acknowledge. Firstly, it was designed as a cross-sectional observational study, so no causal relationship can be drawn. Our results should be further investigated in a large prospective cohort. Secondly, the cross-sectional design may lead to a selection bias, especially with respect to the healthy elderly. However, excluding subjects with histories of cardiovascular diseases might partially decrease the bias. Thirdly, the study population was less likely to be smokers than the general Chinese population. Therefore, caution should be exercised when generalizing the results. Finally, the assessment of CAP with ultrasound may be less reliable than with magnetic resonance imaging or computed tomography. However, considering its convenience and the likelihood of technical improvements, ultrasound still remains the first choice for CAP assessment.

In conclusion, this study found that the total WBC, neutrophil, and monocyte counts are associated with the presence, severity, and types of CAPs in healthy Chinese adults, and these markers may be useful in clinical practice.

## Data Availability Statement

The datasets generated for this study are available on request to the corresponding author.

## Ethics Statement

The studies involving human participants were reviewed and approved by Research Ethical Committee of the First Affiliated Hospital of Zhengzhou University. The patients/participants provided their written informed consent to participate in this study.

## Author Contributions

YX and YL were the project lead for the current study. YZ, WJ, DS, and LZ established the database and extracted data. YL, YC, and CZ conducted data and statistical analysis. YL, YZ, and GC wrote the manuscript. QL and YC provided significant advice on the manuscript. YB and YX reviewed and revised the manuscript. All authors read and approved the final manuscript.

## Conflict of Interest

The authors declare that the research was conducted in the absence of any commercial or financial relationships that could be construed as a potential conflict of interest.
