# Supplementary material for: Association of the Total White Blood Cell, Neutrophils, and Monocytes Count With the Presence, Severity, and Types of Carotid Atherosclerotic Plaque
Source: Front Med (Lausanne). 2020 Jul 21;7:313. doi: 10.3389/fmed.2020.00313 (PMC7385072; doi:10.3389/fmed.2020.00313)
Supplement: Supplementary file 1 [file Data_Sheet_1.docx]

**Table S1**. Covariate-adjusted mean (SEM) total WBC and differential WBC count by the counts of carotid artery plaques

|  | Count of carotid plaque | | | | | | | | | | | | *P* | *P*_-trend_ |
| --- | --- | --- | --- | --- | --- | --- | --- | --- | --- | --- | --- | --- | --- | --- |
|  | 0 | | | 1 | | | 2 | | | ≥3 | | |  |  |
|  | n | mean | SE | n | mean | SEM | n | mean | SEM | n | mean | SEM |  |  |
| Total WBC count，×10^9^/L |  |  |  |  |  |  |  |  |  |  |  |  |  |  |
| Model 1 | 1539 | 6.11 | 0.04 | 517 | 6.30 | 0.07 | 302 | 6.50 | 0.10^**^ | 399 | 6.44 | 0.09^*^ | **0.001** | **0.001** |
| Model 2 | 1327 | 6.14 | 0.05 | 445 | 6.27 | 0.08 | 254 | 6.55 | 0.10^**^ | 337 | 6.47 | 0.10^*^ | **0.001** | **0.001** |
| Neutrophils, ×10^9^/L |  |  |  |  |  |  |  |  |  |  |  |  |  |  |
| Model 1 | 1546 | 3.55 | 0.04 | 522 | 3.78 | 0.06^*^ | 302 | 3.82 | 0.09^*^ | 400 | 3.76 | 0.08 | **0.003** | **0.030** |
| Model 2 | 1333 | 3.55 | 0.04 | 450 | 3.77 | 0.07 | 254 | 3.86 | 0.10^*^ | 338 | 3.78 | 0.09 | **0.006** | **0.021** |
| Neutrophils, % |  |  |  |  |  |  |  |  |  |  |  |  |  |  |
| Model 1 | 1545 | 57.14 | 0.21 | 522 | 58.19 | 0.34 | 301 | 58.28 | 0.45 | 399 | 57.85 | 0.43 | **0.026** | 0.177 |
| Model 2 | 1332 | 56.97 | 0.22 | 450 | 58.17 | 0.36^*^ | 254 | 58.45 | 0.49^*^ | 337 | 58.01 | 0.46 | **0.007** | 0.051 |
| Lymphocytes, ×10^9^/L |  |  |  |  |  |  |  |  |  |  |  |  |  |  |
| Model 1 | 1545 | 1.99 | 0.02 | 522 | 2.00 | 0.04 | 302 | 2.07 | 0.05 | 400 | 2.04 | 0.04 | 0.415 | 0.173 |
| Model 2 | 1332 | 2.01 | 0.02 | 450 | 1.99 | 0.04 | 254 | 2.07 | 0.05 | 338 | 2.03 | 0.05 | 0.615 | 0.407 |
| Lymphocytes, % |  |  |  |  |  |  |  |  |  |  |  |  |  |  |
| Model 1 | 1523 | 32.92 | 0.20 | 519 | 32.01 | 0.32 | 298 | 32.05 | 0.44 | 396 | 32.02 | 0.41 | 0.052 | 0.091 |
| Model 2 | 1311 | 33.11 | 0.22 | 447 | 32.02 | 0.35 | 250 | 31.87 | 0.47 | 334 | 31.78 | 0.44 | **0.008** | **0.015** |
| Monocytes, ×10^9^/L |  |  |  |  |  |  |  |  |  |  |  |  |  |  |
| Model 1 | 1543 | 0.42 | 0.00 | 522 | 0.43 | 0.01 | 301 | 0.44 | 0.01 | 399 | 0.47 | 0.01^***^ | **<0.001** | **<0.001** |
| Model 2 | 1331 | 0.42 | 0.00 | 450 | 0.44 | 0.01 | 253 | 0.45 | 0.01 | 337 | 0.46 | 0.01^***^ | **<0.001** | **<0.001** |
| Monocytes, % |  |  |  |  |  |  |  |  |  |  |  |  |  |  |
| Model 1 | 1544 | 6.89 | 0.08 | 522 | 7.08 | 0.12 | 302 | 6.87 | 0.16 | 400 | 7.10 | 0.15 | 0.437 | 0.490 |
| Model 2 | 1331 | 6.95 | 0.08 | 450 | 7.23 | 0.14 | 254 | 6.86 | 0.18 | 338 | 7.21 | 0.17 | 0.161 | 0.520 |
| Eosinophils, ×10^9^/L | |  |  |  |  |  |  |  |  |  |  |  |  |  |
| Model 1 | 1545 | 0.39 | 0.03 | 521 | 0.36 | 0.04 | 302 | 0.37 | 0.06 | 400 | 0.45 | 0.05 | 0.570 | 0.404 |
| Model 2 | 1332 | 0.42 | 0.03 | 449 | 0.38 | 0.05 | 254 | 0.37 | 0.07 | 338 | 0.52 | 0.06 | 0.295 | 0.230 |
| Eosinophils, % |  |  |  |  |  |  |  |  |  |  |  |  |  |  |
| Model 1 | 1546 | 2.29 | 0.06 | 522 | 2.17 | 0.09 | 302 | 2.07 | 0.13 | 400 | 2.10 | 0.12 | 0.323 | 0.145 |
| Model 2 | 1333 | 2.28 | 0.06 | 450 | 2.12 | 0.10 | 254 | 2.09 | 0.14 | 338 | 2.08 | 0.13 | 0.396 | 0.213 |
| Basophils, ×10^9^/L | |  |  |  |  |  |  |  |  |  |  |  |  |  |
| Model 1 | 1545 | 0.05 | 0.00 | 522 | 0.05 | 0.00 | 301 | 0.04 | 0.00 | 400 | 0.05 | 0.00 | 0.582 | 0.610 |
| Model 2 | 1332 | 0.04 | 0.00 | 450 | 0.04 | 0.00 | 253 | 0.04 | 0.00 | 338 | 0.05 | 0.00 | 0.485 | 0.580 |
| Basophils%, % |  |  |  |  |  |  |  |  |  |  |  |  |  |  |
| Model 1 | 1546 | 0.52 | 0.01 | 522 | 0.51 | 0.01 | 302 | 0.50 | 0.01 | 400 | 0.54 | 0.01 | 0.071 | 0.515 |
| Model 2 | 1333 | 0.52 | 0.01 | 450 | 0.52 | 0.01 | 254 | 0.50 | 0.01 | 338 | 0.54 | 0.01 | 0.363 | 0.592 |

WBC: white blood cells. Bold *P* indicates statistical significance.

Model 1 adjusted for age and BMI.

Model 2 included additional adjustments for systolic blood pressure, diastolic blood pressure, total cholesterol, triglycerides, high-density lipoprotein cholesterol, low-density lipoprotein cholesterol, fasting plasma glucose and uric acid.

*,**,***: *P* ≤ 0.05, *P* ≤ 0.01, *P* ≤ 0.001, compared with group 0 (Bonferroni).

**Table S2**．Covariate-adjusted mean (SEM) total WBC and differential WBC count by the maximal internal carotid plaque thickness (MICPT)

|  | Maximal internal carotid plaque thickness (MICPT) | | | | | | | | | *P* | *P*_-trend_ |
| --- | --- | --- | --- | --- | --- | --- | --- | --- | --- | --- | --- |
|  | Q1-Q2 | | | Q3 | | | Q4 | | |  |  |
|  | n | mean | SE | n | mean | SEM | n | mean | SEM |  |  |
| Total WBC count，×10^9^/L |  |  |  |  |  |  |  |  |  |  |  |
| Model 1 | 1542 | 6.12 | 0.04 | 537 | 6.38 | 0.07^**^ | 679 | 6.40 | 0.07^**^ | **0.001** | **0.001** |
| Model 2 | 1330 | 6.15 | 0.05 | 467 | 6.37 | 0.08^*^ | 566 | 6.42 | 0.07^*^ | **0.005** | **0.004** |
| Neutrophils, ×10^9^/L |  |  |  |  |  |  |  |  |  |  |  |
| Model 1 | 1549 | 3.55 | 0.04 | 540 | 3.82 | 0.06^**^ | 682 | 3.75 | 0.06^*^ | **0.001** | **0.009** |
| Model 2 | 1336 | 3.56 | 0.04 | 470 | 3.83 | 0.07^**^ | 569 | 3.76 | 0.07^*^ | **0.002** | **0.015** |
| Neutrophils, % |  |  |  |  |  |  |  |  |  |  |  |
| Model 1 | 1548 | 57.15 | 0.21 | 540 | 58.38 | 0.34^**^ | 680 | 57.88 | 0.32 | **0.007** | 0.070 |
| Model 2 | 1335 | 56.98 | 0.22 | 470 | 58.39 | 0.36^**^ | 568 | 58.01 | 0.35 | **0.002** | **0.018** |
| Lymphocytes, ×10^9^/L |  |  |  |  |  |  |  |  |  |  |  |
| Model 1 | 1548 | 1.97 | 0.02 | 540 | 2.01 | 0.02 | 682 | 2.04 | 0.02 | 0.083 | **0.029** |
| Model 2 | 1335 | 1.99 | 0.02 | 470 | 2.00 | 0.03 | 569 | 2.03 | 0.03 | 0.358 | 0.155 |
| Lymphocytes, % |  |  |  |  |  |  |  |  |  |  |  |
| Model 1 | 1526 | 32.83 | 0.19 | 535 | 31.81 | 0.31^*^ | 676 | 32.22 | 0.29 | **0.019** | 0.104 |
| Model 2 | 1334 | 33.00 | 0.20 | 465 | 31.76 | 0.33^**^ | 563 | 32.09 | 0.32 | **0.003** | **0.022** |
| Monocytes, ×10^9^/L |  |  |  |  |  |  |  |  |  |  |  |
| Model 1 | 1546 | 0.42 | 0.00 | 540 | 0.43 | 0.01 | 680 | 0.45 | 0.01^***^ | **0.001** | **<0.001** |
| Model 2 | 1334 | 0.42 | 0.00 | 470 | 0.44 | 0.01 | 567 | 0.45 | 0.01^**^ | **0.003** | **0.001** |
| Monocytes, % |  |  |  |  |  |  |  |  |  |  |  |
| Model 1 | 1547 | 6.90 | 0.05 | 540 | 6.84 | 0.08 | 682 | 7.02 | 0.07 | 0.231 | 0.213 |
| Model 2 | 1334 | 6.96 | 0.05 | 470 | 6.98 | 0.08 | 569 | 7.06 | 0.08 | 0.526 | 0.266 |
| Eosinophils, ×10^9^/L |  |  |  |  |  |  |  |  |  |  |  |
| Model 1 | 1548 | 0.40 | 0.03 | 540 | 0.47 | 0.04 | 682 | 0.31 | 0.04 | **0.017** | 0.069 |
| Model 2 | 1335 | 0.43 | 0.03 | 470 | 0.50 | 0.05 | 568 | 0.34 | 0.05 | **0.036** | 0.125 |
| Eosinophils, % |  |  |  |  |  |  |  |  |  |  |  |
| Model 1 | 1549 | 2.29 | 0.06 | 540 | 2.08 | 0.09 | 682 | 2.16 | 0.09 | 0.150 | 0.224 |
| Model 2 | 1336 | 2.28 | 0.06 | 470 | 2.06 | 0.10 | 569 | 2.13 | 0.10 | 0.165 | 0.212 |
| Basophils, ×10^9^/L |  |  |  |  |  |  |  |  |  |  |  |
| Model 1 | 1548 | 0.05 | 0.00 | 540 | 0.04 | 0.00 | 681 | 0.05 | 0.00 | 0.471 | 0.572 |
| Model 2 | 1335 | 0.04 | 0.00 | 470 | 0.04 | 0.00 | 568 | 0.04 | 0.00 | 0.769 | 0.998 |
| Basophils, % |  |  |  |  |  |  |  |  |  |  |  |
| Model 1 | 1549 | 0.52 | 0.01 | 540 | 0.51 | 0.01 | 682 | 0.52 | 0.01 | 0.521 | 0.255 |
| Model 2 | 1336 | 0.52 | 0.01 | 470 | 0.52 | 0.01 | 569 | 0.52 | 0.01 | 0.805 | 0.875 |

WBC: white blood cells. Bold *P* indicates statistical significance.

Model 1 adjusted for age and BMI.

Model 2 included additional adjustments for systolic blood pressure, diastolic blood pressure, total cholesterol, triglycerides, high-density lipoprotein cholesterol, low-density lipoprotein cholesterol, fasting plasma glucose and uric acid.

*,**,***: *P* ≤ 0.05, *P* ≤ 0.01, *P* ≤ 0.001, compared with Q1-Q2 (Bonferroni).

**Table S3**．Covariate-adjusted mean (SEM) total WBC and differential WBC count by plaque score

|  | Plaque score | | | | | | | | | *P* | *P*_-trend_ |
| --- | --- | --- | --- | --- | --- | --- | --- | --- | --- | --- | --- |
|  | Q1-Q2 | | | Q3 | | | Q4 | | |  |  |
|  | n | mean | SE | n | mean | SEM | n | mean | SEM |  |  |
| Total WBC count, ×10^9^/L |  |  |  |  |  |  |  |  |  |  |  |
| Model 1 | 1542 | 6.11 | 0.04 | 532 | 6.29 | 0.07 | 684 | 6.48 | 0.07^***^ | **<0.001** | **<0.001** |
| Model 2 | 1330 | 6.14 | 0.05 | 458 | 6.24 | 0.08 | 575 | 6.54 | 0.08^***^ | **<0.001** | **<0.001** |
| Neutrophils, ×10^9^/L |  |  |  |  |  |  |  |  |  |  |  |
| Model 1 | 1549 | 3.55 | 0.04 | 537 | 3.77 | 0.06^**^ | 685 | 3.79 | 0.06^**^ | **0.001** | **0.002** |
| Model 2 | 1336 | 3.55 | 0.04 | 463 | 3.76 | 0.07^*^ | 576 | 3.83 | 0.07^**^ | **0.002** | **0.001** |
| Neutrophils, % |  |  |  |  |  |  |  |  |  |  |  |
| Model 1 | 1548 | 57.16 | 0.21 | 537 | 58.34 | 0.33^**^ | 683 | 57.91 | 0.33 | **0.009** | 0.069 |
| Model 2 | 1335 | 56.98 | 0.22 | 463 | 58.34 | 0.36^**^ | 575 | 58.05 | 0.35^*^ | **0.003** | **0.016** |
| Lymphocytes, ×10^9^/L |  |  |  |  |  |  |  |  |  |  |  |
| Model 1 | 1548 | 1.97 | 0.02 | 537 | 1.98 | 0.02 | 685 | 2.06 | 0.02^**^ | **0.011** | **0.003** |
| Model 2 | 1335 | 1.99 | 0.02 | 463 | 1.97 | 0.03 | 576 | 2.06 | 0.03 | **0.020** | **0.017** |
| Lymphocytes, % |  |  |  |  |  |  |  |  |  |  |  |
| Model 1 | 1526 | 32.83 | 0.19 | 534 | 31.89 | 0.31^*^ | 677 | 32.16 | 0.30 | 0.026 | 0.079 |
| Model 2 | 1314 | 33.01 | 0.20 | 460 | 31.88 | 0.33^*^ | 568 | 31.98 | 0.32^*^ | **0.004** | **0.011** |
| Monocytes, ×10^9^/L |  |  |  |  |  |  |  |  |  |  |  |
| Model 1 | 1546 | 0.42 | 0.00 | 537 | 0.43 | 0.01 | 683 | 0.46 | 0.01^***^ | **<0.001** | **<0.001** |
| Model 2 | 1334 | 0.42 | 0.00 | 563 | 0.43 | 0.01 | 574 | 0.46 | 0.01^***^ | **<0.001** | **<0.001** |
| Monocytes, % |  |  |  |  |  |  |  |  |  |  |  |
| Model 1 | 1547 | 6.90 | 0.05 | 537 | 6.87 | 0.08 | 685 | 7.00 | 0.08 | 0.467 | 0.319 |
| Model 2 | 1334 | 6.96 | 0.05 | 463 | 6.99 | 0.08 | 576 | 7.06 | 0.08 | 0.599 | 0.312 |
| Eosinophils, ×10^9^/L | |  |  |  |  |  |  |  |  |  |  |
| Model 1 | 1548 | 0.40 | 0.03 | 536 | 0.36 | 0.04 | 685 | 0.41 | 0.04 | 0.714 | 0.825 |
| Model 2 | 1335 | 0.42 | 0.03 | 462 | 0.38 | 0.05 | 576 | 0.45 | 0.05 | 0.604 | 0.609 |
| Eosinophils, %, |  |  |  |  |  |  |  |  |  |  |  |
| Model 1 | 1549 | 2.30 | 0.06 | 537 | 2.14 | 0.09 | 685 | 2.11 | 0.09 | 0.173 | 0.098 |
| Model 2 | 1336 | 2.28 | 0.06 | 463 | 2.10 | 0.10 | 576 | 2.09 | 0.10 | 0.185 | 0.129 |
| Basophils, ×10^9^/L | |  |  |  |  |  |  |  |  |  |  |
| Model 1 | 1548 | 0.05 | 0.00 | 537 | 0.05 | 0.00 | 684 | 0.05 | 0.00 | 0.560 | 0.378 |
| Model 2 | 1335 | 0.04 | 0.00 | 463 | 0.04 | 0.00 | 575 | 0.04 | 0.00 | 0.745 | 0.956 |
| Basophils%, % |  |  |  |  |  |  |  |  |  |  |  |
| Model 1 | 1549 | 0.52 | 0.01 | 537 | 0.51 | 0.01 | 685 | 0.52 | 0.01 | 0.537 | 0.614 |
| Model 2 | 1336 | 0.52 | 0.01 | 463 | 0.52 | 0.01 | 576 | 0.52 | 0.01 | 0.870 | 0.778 |

WBC: white blood cells. Bold *P* indicates statistical significance.

Model 1 adjusted for age and BMI.

Model 2 included additional adjustments for systolic blood pressure, diastolic blood pressure, total cholesterol, triglycerides, high-density lipoprotein cholesterol, low-density lipoprotein cholesterol, fasting plasma glucose and uric acid.

*,**,***: *P* ≤ 0.05, *P* ≤ 0 .01, *P* ≤ 0.001, compareed with Q1-Q2 (Bonferroni).

**Table S4**. Covariate-adjusted mean (SEM) total WBC and differential WBC count by the composition of carotid artery plaque

|  | Carotid plaque composition | | | | | | | | | | | | | |  |  |
| --- | --- | --- | --- | --- | --- | --- | --- | --- | --- | --- | --- | --- | --- | --- | --- | --- |
|  | None | | | Echolucent | | | Echogenic | | | Heterogeneous | | | Polytype | | | *P* |
|  | n | mean | SE | n | mean | SE | n | mean | SEM | n | mean | SEM | n | mean | SEM |  |
| Total WBC count, ×10^9^/L |  |  |  |  |  |  |  |  |  |  |  |  |  |  |  |  |
| Model 1 | 1539 | 6.12 | 0.04 | 486 | 6.42 | 0.08^**^ | 20 | 6.31 | 0.37 | 341 | 6.29 | 0.09 | 372 | 6.44 | 0.091^*^ | **0.002** |
| Model 2 | 1327 | 6.15 | 0.05 | 415 | 6.44 | 0.08^*^ | 19 | 5.83 | 0.37 | 292 | 6.29 | 0.10 | 310 | 6.46 | 0.10 | **0.005** |
| Neutrophils, ×10^9^/L |  |  |  |  |  |  |  |  |  |  |  |  |  |  |  |  |
| Model 1 | 1546 | 3.55 | 0.04 | 490 | 3.88 | 0.07^***^ | 20 | 3.72 | 0.32 | 342 | 3.65 | 0.08 | 373 | 3.75 | 0.08 | **0.001** |
| Model 2 | 1333 | 3.56 | 0.04 | 419 | 3.91 | 0.07^***^ | 19 | 3.36 | 0.34 | 293 | 3.64 | 0.09 | 311 | 3.78 | 0.09 | **0.001** |
| Neutrophils, % |  |  |  |  |  |  |  |  |  |  |  |  |  |  |  |  |
| Model 1 | 1545 | 57.18 | 0.21 | 489 | 58.78 | 0.35^**^ | 20 | 57.88 | 1.72 | 342 | 57.26 | 0.43 | 372 | 57.91 | 0.43 | **0.003** |
| Model 2 | 1332 | 57.00 | 0.22 | 418 | 58.80 | 0.37 | 19 | 57.20 | 1.75 | 293 | 57.23 | 0.46 | 311 | 58.21 | 0.46 | **0.001** |
| Lymphocytes, ×10^9^/L |  |  |  |  |  |  |  |  |  |  |  |  |  |  |  |  |
| Model 1 | 1545 | 1.99 | 0.02 | 490 | 2.00 | 0.04 | 20 | 1.94 | 0.18 | 342 | 2.04 | 0.05 | 373 | 2.05 | 0.04 | 0.692 |
| Model 2 | 1332 | 2.01 | 0.02 | 419 | 2.01 | 0.04 | 19 | 1.86 | 0.19 | 293 | 2.05 | 0.05 | 311 | 2.03 | 0.05 | 0.864 |
| Lymphocytes, % |  |  |  |  |  |  |  |  |  |  |  |  |  |  |  |  |
| Model 1 | 1523 | 32.89 | 0.20 | 486 | 31.55 | 0.336^**^ | 20 | 31.48 | 1.65 | 342 | 32.76 | 0.42 | 366 | 32.07 | 0.41 | **0.011** |
| Model 2 | 1311 | 33.08 | 0.22 | 415 | 31.47 | 0.363^**^ | 19 | 32.14 | 1.69 | 293 | 32.80 | 0.45 | 304 | 31.77 | 0.45 | **0.002** |
| Monocytes, ×10^9^/L |  |  |  |  |  |  |  |  |  |  |  |  |  |  |  |  |
| Model 1 | 1543 | 0.42 | 0.00 | 490 | 0.43 | 0.01 | 20 | 0.49 | 0.04 | 342 | 0.45 | 0.01 | 371 | 0.46 | 0.009^**^ | **0.001** |
| Model 2 | 1331 | 0.42 | 0.00 | 419 | 0.44 | 0.01 | 19 | 0.47 | 0.03 | 293 | 0.45 | 0.01 | 309 | 0.45 | 0.008^*^ | **0.011** |
| Monocytes, % |  |  |  |  |  |  |  |  |  |  |  |  |  |  |  |  |
| Model 1 | 1544 | 6.89 | 0.08 | 490 | 6.96 | 0.13 | 20 | 7.79 | 0.62 | 342 | 7.12 | 0.16 | 373 | 7.01 | 0.15 | 0.492 |
| Model 2 | 1331 | 6.95 | 0.08 | 419 | 7.11 | 0.14 | 19 | 7.92 | 0.66 | 293 | 7.18 | 0.17 | 311 | 7.07 | 0.17 | 0.446 |
| Eosinophils, ×10^9^/L | |  |  |  |  |  |  |  |  |  |  |  |  |  |  |  |
| Model 1 | 1545 | 0.40 | 0.03 | 490 | 0.35 | 0.04 | 20 | 0.50 | 0.22 | 341 | 0.38 | 0.06 | 373 | 0.44 | 0.05 | 0.723 |
| Model 2 | 1332 | 0.42 | 0.03 | 419 | 0.38 | 0.05 | 19 | 0.48 | 0.24 | 292 | 0.42 | 0.06 | 311 | 0.48 | 0.06 | 0.794 |
| Eosinophils, % |  |  |  |  |  |  |  |  |  |  |  |  |  |  |  |  |
| Model 1 | 1546 | 2.29 | 0.06 | 490 | 2.16 | 0.10 | 20 | 1.92 | 0.48 | 342 | 2.11 | 0.12 | 373 | 2.11 | 0.12 | 0.504 |
| Model 2 | 1333 | 2.28 | 0.06 | 419 | 2.18 | 0.11 | 19 | 1.89 | 0.49 | 293 | 2.02 | 0.13 | 311 | 2.07 | 0.13 | 0.393 |
| Basophils, ×10^9^/L | |  |  |  |  |  |  |  |  |  |  |  |  |  |  |  |
| Model 1 | 1545 | 0.05 | 0.00 | 489 | 0.05 | 0.00 | 20 | 0.06 | 0.02 | 342 | 0.04 | 0.00 | 373 | 0.05 | 0.00 | 0.392 |
| Model 2 | 1332 | 0.04 | 0.00 | 418 | 0.04 | 0.00 | 19 | 0.07 | 0.02 | 293 | 0.04 | 0.00 | 311 | 0.05 | 0.00 | 0.392 |
| Basophils, % |  |  |  |  |  |  |  |  |  |  |  |  |  |  |  |  |
| Model 1 | 1546 | 0.52 | 0.01 | 490 | 0.50 | 0.01 | 20 | 0.56 | 0.05 | 342 | 0.51 | 0.01 | 373 | 0.54 | 0.01 | 0.149 |
| Model 2 | 1333 | 0.52 | 0.01 | 419 | 0.51 | 0.01 | 19 | 0.58 | 0.05 | 293 | 0.51 | 0.01 | 311 | 0.54 | 0.01 | 0.153 |

WBC: white blood cells. Bold *P* indicates statistical significance.

Model 1 adjusted for age and BMI.

Model 2 included additional adjustments for systolic blood pressure, diastolic blood pressure, total cholesterol, triglycerides, high-density lipoprotein cholesterol, low-density lipoprotein cholesterol, fasting plasma glucose and uric acid.

*,**,***: *P* ≤ 0.05, *P* ≤ 0.01, *P* ≤ 0.001, compared with none (Bonferroni).


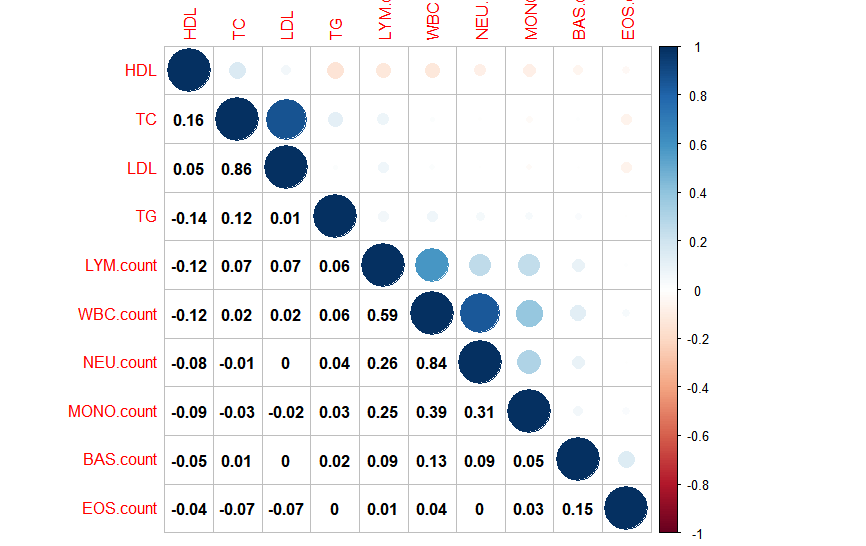


Figure S1. Pearson analysis of the relationship between leukocytes and blood lipid profiles. WBC.count = white blood cell count; NEU.count = neutrophils count; LYM.count = lymphocytes count; Mono.count = monocytes count; BAS.count = basophils count; EOS.count = eosinophils count.


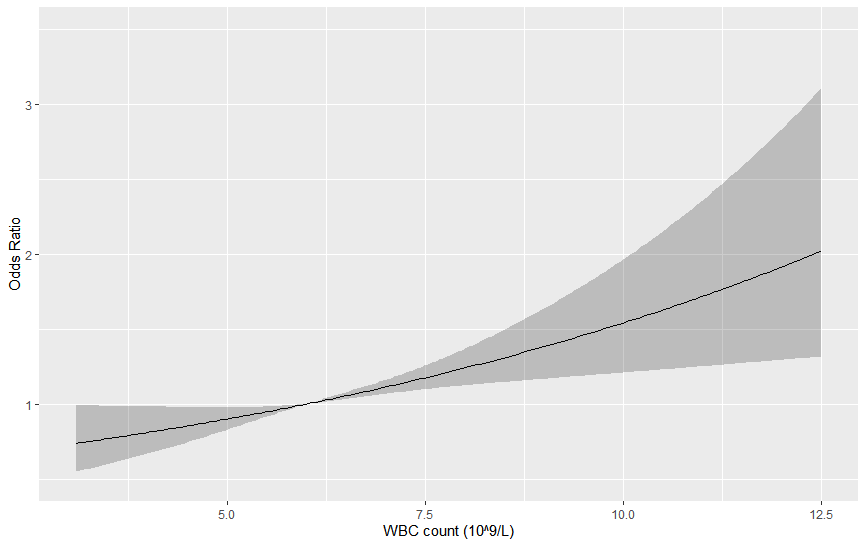


Figure S2. Relationship between the total WBC count and the presence of CAP modelled with restricted cubic spline regression


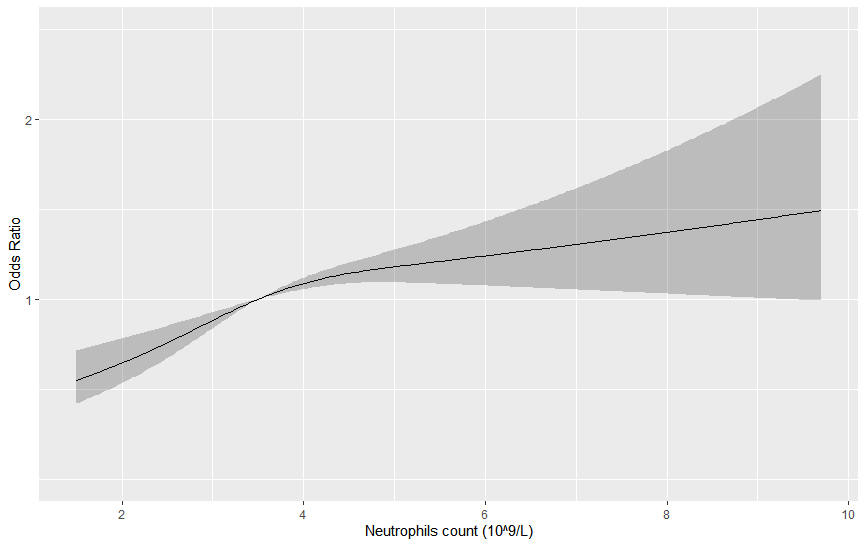


Figure S3. Relationship between the neutrophil count and the presence of CAP modelled with restricted cubic spline regression


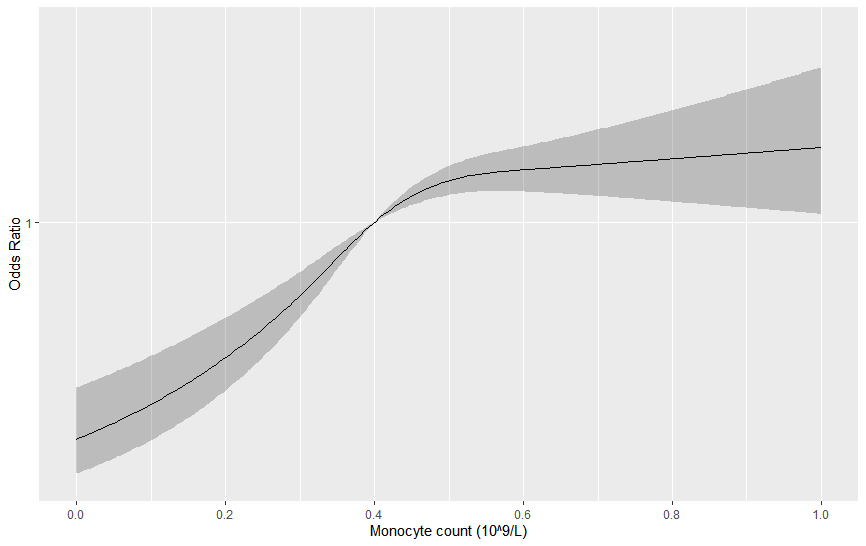


Figure S4. Relationship between the monocyte count and the presence of CAP modelled with restricted cubic spline regression
